# Sentinel lymph node biopsy for high-thickness cutaneous squamous cell carcinoma

**DOI:** 10.1007/s00403-020-02082-1

**Published:** 2020-05-08

**Authors:** Lukas Kofler, Katrin Kofler, Claudia Schulz, Helmut Breuninger, Hans-Martin Häfner

**Affiliations:** Department of Dermatology and Center for Dermatologic Surgery, Eberhard-Karls University of Tuebingen, Liebermeisterstraße 25, 72076 Tübingen, Germany

**Keywords:** Squamous cell carcinoma, Dermatologic surgery, Sentinel lymph node biopsy, Oncology

## Abstract

Squamous cell carcinomas are among the most common skin tumors and show a risk of metastasis depending on various factors such as tumor thickness, localization, histological subtype and immune status of the patient. Sentinel lymph node biopsy (SLNB) SLNB represents a possibility for assessing the locoregional lymph node status. In this study, the role of the SLNB in lymph node status and survival was analyzed. Retrospectively, 720 patients with high-risk squamous cell carcinoma (tumor thickness > 5 mm) were examined. 150 patients agreed to SLNB, 570 patients did not undergo histologic confirmation of lymph node status and were included directly in follow-up. In 101 patients, a sentinel lymph node was successfully marked and extirpated, followed by regular follow-up examinations.

A total of 11.11% of the patients showed lymph node metastasis in the course of their treatment, with no difference in the proportion of patients in the SLNB group (11.9%) and the observation group (11.4%) (*p* = 0.873). The proportion of distant metastasis also did not differ between the groups (*p* = 0.898). In 3.96% of the patients in the SLNB group, a metastasis was found in the sentinel lymph node. Tumor-specific death was observed in 7.14% of the patients in the SLNB group and 4.74% in the observation group (*p* = 0.269). Although SLNB is a principally suitable method for determining lymph node status, the available data do not provide any benefit regarding further metastasis or tumor-specific survival.

## Introduction

Cutaneous squamous cell carcinomas (cSCC) are the second most frequent skin tumors [23]. Thereby about 20% of all non-melanocytic tumors are counted as cSCC, whereas an increased number of cases can be expected in the future decades [20]. A recent epidemiological study expects the incidence of non-melanoma skin cancer to double by the year 2030 [19]. This is also of considerable importance, as cSCC has not only metastatic potential but also shows aggressive courses in immunosuppressed patients [26, 29]. Various factors contribute to the risk of progressive disease in cSCC, including tumor parameters such as tumor thickness, histological subtype and tumor localization, as well as patient-related factors such as the presence of immunosuppression [5, 32, 33]. Tumor thickness plays an important role for the risk of local recurrence after excision as well as for metastasis [33]. Brantsch et al. were able to show that metastasis only occurred in patients with a tumor thickness of at least 2 mm [5].

The therapy of first choice for cutaneous cSCC is complete surgical removal, whereby micrographically controlled surgery is of great importance [6, 14, 22]. Therefore, specific techniques such as Mohs surgery or 3D histology can be used to ensure greater safety regarding the complete removal of the tumor as well as to achieve better esthetic results by minimizing the impact on healthy tissue [9, 22]. Since cSCC primarily causes lymphatic metastases, a sentinel lymph node biopsy (SLNB) is a method to evaluate the status of locoregional lymph nodes [4, 17, 25, 27]. SLNB is well established for melanoma, breast carcinoma and certain other tumor entities and has also been in use in cSCC for years [3, 8, 13, 17]. The significance of SLNB in cSCC has been discussed controversially, whereby the indication is usually made based on an increased risk for metastasis ('high-risk patients'). Various risk factors associated with a higher risk of metastasis are used for this purpose, including tumor thickness, tumor diameter greater than 2 cm, degree of differentiation or histological subtype, as well as tumor localization and immune status [31, 33]. According to the German guideline, an SLNB can be considered if an increased risk of metastatic disease is expected for a specific patient [6].

The aim of the present study was to evaluate the role of SLNB in high-risk patients with a tumor thickness of at least 5 mm and to analyze the impact of this procedure regarding lymph node metastasis and survival.

## Methods

### Patients and study design

Patients with cSCC and a tumor thickness of at least 5 mm who underwent surgery between 1999 and 2014 at the Department of Dermatology at the University Hospital of Tübingen were included. The diagnosis was histologically confirmed in all patients and the primary tumor was completely resected whereby 3D histology was used. The tumor thickness cutoff is based on published data on risk stratification [5].An increased metastasis rate has been described for tumors with a thickness of more than 2 mm, increasing with thickness. The tumor thickness of 5 mm was also chosen to take into account the risk that the tumor thickness might be underestimated due to tumor factors such as ulceration or for technical reasons in histological processing. Other risk factors such as immune status of the patient, histological subtype or localization of the tumor were considered in individual cases; however, only patients with a tumor thickness of more than 5 mm were included in the present analysis.

All tumors included in this analysis were classified as 'high risk' according to German guidelines and SLNB was therefore indicated in conformity with the existing guideline at the time of surgery. Several risk factors for locoregional progress and tumor-specific survival were identified and described in the German guideline. These are tumor diameter, tumor thickness and depth of infiltration, as well as the degree of differentiation and perineural infiltration. In addition, the localization of the tumor (e.g., on the lip) and the presence of immunosuppression are also listed in the guidelines.

SLNB was offered to all patients, whereby the decision for this intervention was made individually by the patients after a detailed information was provided by an experienced physician. The procedures were performed under tumescent local anesthesia (TLA); analgosedation was added if necessary. Patients who underwent SLNB were subsequently included in regular follow-up with clinical and sonographic controls. Patients who chose not to have SLNB were also included in regular clinical and sonographic follow-up. Data analysis was performed retrospectively. Therefore, patients were categorized as an observation group and an SLNB group. The patients were analyzed retrospectively. The patient's decision regarding SLNB or clinical and sonographic controls was made after a detailed consultation with an experienced physician in the context of clinical routine in our hospital. The analysis of the patients in both the SLNB group and the observation group was carried out retrospectively based on the individual therapy decision of the patients.

Patients underwent close follow-up if the sentinel lymph node remained unmarked or the procedure had to be terminated intraoperatively to avoid iatrogenic vascular or nerve injury due to the location of the lymph node.

The follow-up examinations were carried out according to a standardized protocol based on recommendations of the German guideline. Every 6 months, patients were clinically examined and sonography of the locoregional lymph node stations was performed. Follow-up examinations were carried out for 3 years, after which, depending on individual risk factors (such as immune status, secondary tumors and primary tumor parameters), either the follow-up intervals were maintained or changed to annual visits in case of no relapse and a low-risk profile.

In patients who were immunosuppressed due to organ transplantation, follow-up examinations were carried out at shorter intervals and patients were monitored in a specialized department of our hospital. Shorter individual follow-up intervals were also defined for patients at higher risk, such as those with diagnosed leukemia.

The current study was approved by the Ethics Commission of the University of Tübingen (Project Number: 706/2017BO2).

### Surgical approach

On the day of surgery, technetium-99 was injected peritumorally or around the resection scar at the Department for Nuclear Medicine. Immediately before surgery, a gamma probe collimated for technetium-99 (Neoprobe GDS, Devicor Medical; Norderstedt) was used to verify the position of the sentinel node. Additionally, 0.5–1.0 mL of patent blue-dye solution (acid blue 3) was injected intradermally.

The technetium-99 signal was used to locate the lymph node. After skin incision and preparation, the sentinel lymph node was surgically removed from surrounding structures. Blood and lymph vessels were sealed using bipolar forceps. Surgery was discontinued, if no clear signal was detectable or the extirpation of the sentinel lymph node was not possible due to endangerment of vascular or nerve structures.

### Pathological processing of the sentinel lymph node

The specimen of the sentinel lymph node was fixed in formalin. Further microscopic investigation was performed at the University Institute of Pathology by experienced senior physicians. The specimens were stained with hematoxylin/eosin stain as well as cytokeratin-5/6 if required. According to a standard operating procedure implemented at the University Institute of Pathology, sentinel lymph nodes were processed in two sections with two tissue slides per section and stained with hematoxylin/eosin. Further immune staining was performed on an individual basis if the hematoxylin/eosin-stained section alone did not allow a precise diagnosis. Deeper incisions were also made individually if one of the board-certified pathologists decided this to be necessary in the individual case.

### Statistical analysis

All data collected were analyzed using JMP (SAS Institute Cary/NC, USA). Clinical, histopathological and demographic features were statistically evaluated. Numerical variables were described by the mean value. The Wilcoxon–Mann–Whitney test and the two-sided Chi-square-test were used for the analysis. ANCOVA analyses were performed where necessary for covariance analysis.

Survival analyses were one sided according to the study protocol. *p* values below 0.05 were considered statistically significant.

## Results

### Description of the patient cohort

**Fig. 1:**
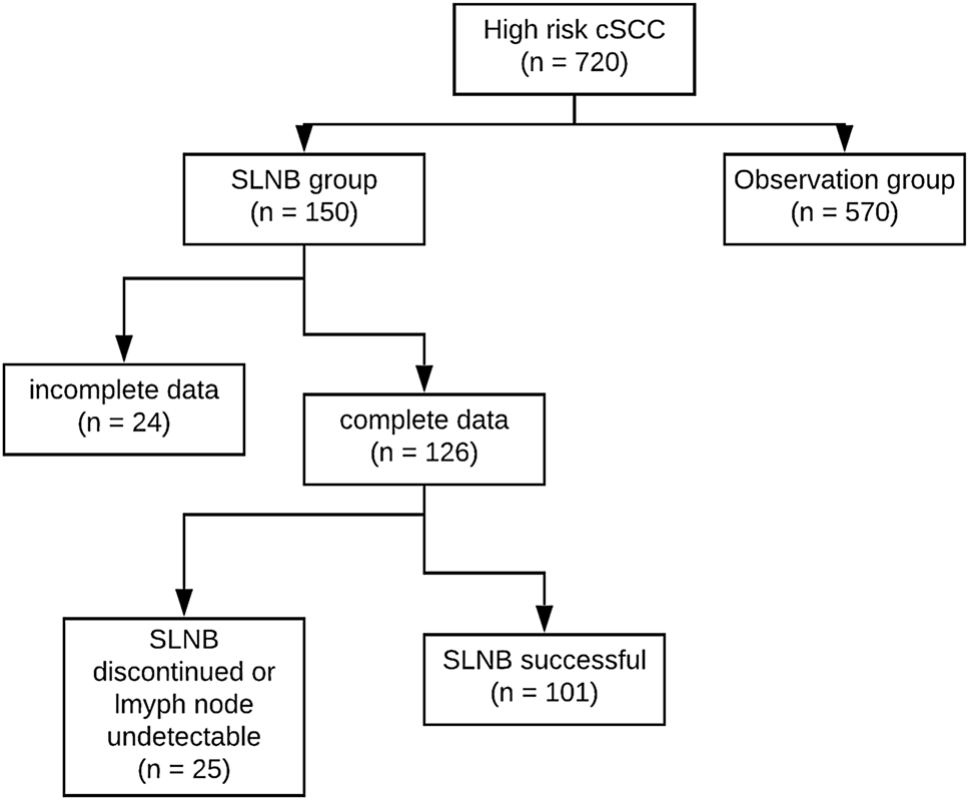
Schematic representation of the groups (SLNB group and observation group)

720 patients with cutaneous squamous cell carcinoma and a vertical tumor thickness of at least 5 mm were included. After individual information of each patients, 150 patients received SLNB (SLNB group) and 570 were closely monitored clinically and sonographically without SLNB (observation group). Incomplete data sets were found in 24 patients of the SLNB group, which led to their exclusion from further analysis (see Fig. 1). In another 25 patients, it was not possible to detect the sentinel lymph node pre-, or intraoperatively or the procedure was discontinued to avoid endangering vascular or nerve structures. Thus, in 101 patients a sentinel lymph node was removed and was therefore available for pathological workup ('technically successful SLNB'; see Fig. 1).

The median follow-up was 2.92 years in the SLNB group and 2.82 years in the observation group. Furthermore, patients of both groups did not differ with regard to the average tumor diameter, safety distance of primary excision, tumor differentiation of the primary tumor or the proportion of desmoplastic squamous cell carcinoma. The diameters of the enclosed tumors ranged between 31.92 mm and 36.1 mm in the SLNB group and between 30.95 mm and 39.15 mm in the observation group.

The SLNB group showed a higher average tumor thickness (*p* = 0.001), a higher proportion of male patients (*p* < 0.001) and of immunosuppressed patients (*p* < 0.001). In contrast, a higher mean patient age was found in the observation group (*p* < 0.001) (see Table 1).

**Table 1:** Patient characteristics

	SLNB group (*n* = 150)	Observation group (*n* = 570)
Maximum tumor diameter (mm)	35.05	34.0
Tumor thickness (mm)	8.72	7.9
Mean safety margin at excision of primary tumor (mm)	6.65	6.37
Desmoplastic cSCC	25 (19.84%)	124 (21.75%)
Immunosuppression	34 (22.67%)	79 (13.86%)
Follow-up (years; median)	2.92	2.82
Age at time of diagnosis	71.78	81.04
Time to local recurrence (years; median)	1.26	1.03
Sex (male/female)	84%/16%	66.3%/33.7%

cSCC were found in 27 body sites, with the head and neck area being the most frequent localization in both the observation group (87.19%) and the SLNB group (80.67%). With regard to distribution, there was no difference for the SLNB and observation groups. Of all patients with evidence of tumor cells in the sentinel lymph node, the cSCC was located on their head or face region in 75% (*n* = 3) and on the upper limb in only 25% (*n* = 1).

### Local recurrence

Local recurrence was found in 11.67% (*n* = 84) of all patients in this study. There was a significant increase in local recurrence in patients with desmoplastic cSCC (23.49%) compared to patients with non-desmoplastic cSCC (8.58%; *p* < 0.001). Local recurrence was more frequent in the SLNB group (19.84%) compared to the observation group (10.35%; *p* = 0.003). However, there was no difference between the groups in the time to local recurrence (SLNB group mean 1.26 years, observation group mean 1.03 years; *p* = 0.94). An incomplete removal of the tumor was highly significantly associated with local recurrence (*p* < 0.001).

### Metastasis

Overall, 11.11% of the patients (*n* = 80) showed metastasis of the locoregional lymph nodes. 11.9% of patients in the SLNB group and 11.4% of patients in the observation group developed metastases (*p* = 0.873). Patient age (*p* < 0.01), desmoplasia (*p* < 0.01) and tumor thickness of the primary tumor (*p* < 0.01) were found to be highly significant parameters for the development of lymph node metastasis.

The probability of developing metastases did not differ between patients with and without tumor detection in the sentinel lymph node. However, patients with desmoplastic cSCC developed metastases more frequently than patients with non-desmoplastic cSCC (*p* = 0.027). Further, an increased risk for the development of metastasis was noted for tumors located at the lips or ears (*p* < 0.001).

Among the 101 patients who successfully underwent SLNB, metastasis in the sentinel lymph node was detected in 4ur patients (3.96%). Three of these patients received a complete dissection of the lymph node, whereby tumor-free lymph nodes were detected in all patients. One patient underwent irradiation of the primary tumor and locoregional lymph nodes immediately after positive SLNB. This decision was made based on rapid tumor growth in accordance with the interdisciplinary tumor board of Tübingen University Hospital.

Of all patients developing locoregional metastases, 90.9% showed tumor-free sentinel lymph nodes. Distant metastasis occurred in 1.58% of patients in the SLNB group and 1.75% of patients in the observation group (*p* = 0.898).

### Survival

Within the follow-up of this study, 56.5% of the included patients died. However, only 11.2% of all deaths were tumor specific. In the SLNB group, more tumor-specific deaths were noticed (7.14%) than in the observation group (4.74%), whereby this relationship was not statistically significant.

## Discussion

The extirpation of a sentinel lymph node after preoperative labeling using a radioactive isotope is an established procedure in dermatologic surgery. It is used particularly for detailed nodal diagnosis of melanoma, but also for other tumors such as cSCC [2, 17, 25, 34]. It was speculated that early detection of subclinical metastases could possibly have an impact on survival. However, this is not supported by the available data. Prospective studies are needed to further clarify this point.

A systematic review by Ross and Schmults showed that SLNB is a possible option for diagnosing lymph node metastases of cSCC with low morbidity [30]. However, a high proportion of anogenital squamous cell carcinoma was included in this study, limiting the applicability of the data to cSCC. In addition, no systematic datasets, but mainly case series were available to the authors. In a retrospective analysis by Krediet et al., only a low sensitivity of SLNB was found regarding the development of lymph node metastases [17]. Both Navarette-Dechent et al. and Allen et al. indicate that SLNB has a high negative predictive value with regard to lymph node metastasis. Both studies could not, however, define a subgroup of patients with cSCC who clearly benefit from SLNB [4, 25].

In the present study, a positive sentinel lymph node was found in 3.96% of the patients. Renzi et al. and Rastrelli et al. [27, 28] reported a comparable proportion of positive sentinel lymph nodes. Locoregional lymph node metastasis was found in 11.11% of our patients, which is comparable to data on head and neck melanomas [18].

It has been shown that in patients without clinically detectable lymph node metastases, lymph node sonography is the imaging modality of choice in preference to CT or MRI [10]. It was suggested by Fox et al. that in high-risk patients without clinically detectable metastases, a sonography of the regional lymph nodes should be performed and in case of a negative result SLNB might be considered, which is also reflected in the American guideline [10]. The importance of lymph node sonography is also evident in analogy to data from melanoma patients, although it should be noted that cSCC leads to a considerably lower proportion of nodal and distant metastases.

Our study demonstrated that age, desmoplasia and tumor thickness are highly significant risk factors for the development of lymph node metastases in the further course of the disease. Schmitt et al. further demonstrated that cSCC with a diameter greater than 20 mm is more likely associated with a positive SLNB result [31]. These factors should be considered in the decision regarding SLNB and generally in the planning of follow-up care. Regular and close follow-up visits are of utmost importance, especially for high-risk patients.

Through the use of immunotherapy in an adjutant setting, SLNB has taken on a new significance in melanoma. A similar development might be possible with corresponding data on immunotherapy in cSCC patients. Therefore, it seems important to re-evaluate the role of SLNB in the light of potential new treatment options in the future. However, sonographic controls are a suitable and non-invasive alternative for the early detection of metastases. Particularly due to the fact that no significant difference was found between the SLNB group and the observation group, ongoing surveillance and ultrasound detection seem to be a reasonable strategy.

The proportion of patients who developed distant metastasis did not differ between the study groups. In addition, 90.9% of patients with metastases showed a tumor-free sentinel lymph node. Our results reflect the recently published data of Jansen et al. which showed that no prognosis for the development of distant metastases can be made based on the SLNB result [16]. In this study, an increased risk for metastasis was demonstrated for positive SLNB [16]. In our cohort, one out of four patients with positive SLNB showed metastasis.

The high proportion of patients with false-negative SLNB results, i.e., lymph node metastasis in the course of initial negative SLNB, can be explained by various factors. The complex lymphatic drainage as well as the anatomical proximity of the tumor and sentinel lymph node at the head and neck area results in a technically difficult detection of the sentinel node [15]. Furthermore, the lymphatic drainage can be altered by previous surgeries. A particular problem is the frequent use of local flaps for defect closure for tumors located on the face. Local flaps typically result in relevant changes of the lymphatic drainage. In addition to these points, pathological workup must also be considered as a possible influencing factor for the false negative rate. The lymph nodes were processed according to a standardized protocol at the University Institute of Pathology, for which two sections per lymph node were prepared. A higher number of sections and smaller gaps between sections might reduce the false negative rate and should be implemented in study protocols for prospective trials.

Analogously, the highest false negative rates for SLNB have been shown for head and neck melanomas compared to other localizations [18].

SLNB can be a challenge for both the surgeon and the pathologist. This is particularly true for the surgeon in the case of cervical SLNB, where, as shown, most SLNBs for cSCC are performed. In addition to a natural learning curve for the surgeon, the localization of the lymph nodes near large vessels or nerve structures is particularly challenging. Due to the complex lymph drainage in the head and neck region, atypical localization of the sentinel lymph node is also possible. Especially for the cervical region, an individual surgical approach is required, which requires considerable experience and appropriate training of the surgeon. In addition, due to the limited space between tumor site and sentinel lymph node in the head and neck region, detection with the gamma probe can be difficult due to diffuse radiation or signal superposition.

Pathological methods are also important for the result and its interpretation. In particular, the number of sections through the lymph node and the staining performed are of great importance for the accuracy of the result. In addition to HE staining, immunohistochemical staining plays a decisive role and, like the number of slices, should be included in a standard protocol and reported with each finding. Overall, a standardization of both the surgical approach and further processing is necessary.

Patients with desmoplastic cSCC showed significantly more local recurrences (*p* < 0.001) in the current study, whereby the proportion of 23.49% is comparable to the data of Breuninger et al., who showed local recurrence in 27.3% [7]. Desmoplastic cSCCs have been associated with a higher proportion of lymph node metastases, which is also reflected in our data [7]. In accordance with the literature, our study also found an increased risk of metastasis for tumors located on the lip or ears [29, 33]. Perineural invasion has been identified as a risk factor for local recurrence, metastasis and tumor-specific death. There is a significant increase in the risk of local recurrence with the detection of perineural invasion, whereby the diameter of the involved nerve is highly relevant with the relative risk increasing with larger diameter [33]. A meta-analysis by Thompson et al. showed that the relative risk for the occurrence of metastases was lower for perineural invasion (2.95) than for local recurrence (4.30) and tumor-specific death (4.06) [33].

The tumor thickness was chosen as the major criterion for risk stratification analogous to the data of Brantsch et al. and tumors with a tumor thickness of less than 5 mm were not included in this analysis [5]. However, all tumors included showed a diameter of more than 20 mm, which corresponds to a tumor stage of at least T2 according to the current AJCC classification [24].

Whether the SLNB status has an influence on the survival of patients with clinically or sonographically negative lymph nodes cannot be answered based on evidence at present. In a recent systematic review by Fox et al., the authors point out that SLNB is a diagnostic option, but no convincing results have been obtained yet [10]. SLNB in patients with cSCC must therefore be discussed individually based on the presence of risk factors such as tumor thickness, tumor diameter and desmoplasia. Ahadiat et al. demonstrated that SLNB is underutilized for high-risk tumors [1]. Based on the available data, SLNB should only be considered for patients with high-risk cSCC and should be assessed individually in each case. Considering the associated morbidity, SLNB should be preferred over prophylactic lymph node dissection.

Although more than half of all patients died within the follow-up, only 11.2% tumor-specific deaths were observed. In the observation group, significantly more patients died of other causes of death, whereby patients in this group were on average older than in the SLNB group. Both, the proportion of individuals with advanced age with comorbidities and cSCC-patients will increase within the next years in industrial nations [19]. Therapy decisions in geriatric patients with cSCC should ideally be based on a geriatric examination and the individual situation of the patients and also consider minimally invasive therapy options [12, 21].

## Limitations

A relevant limitation is the study design as a retrospective analysis without randomization.

The classification of the included cSCC as 'high-risk tumors' was made analogously to the stratification by tumor thickness published by Brantsch et al. [5]. For this reason, the present study included patients with a tumor thickness of 5 mm or more and did not include any other tumors at high-risk locations such as ears or lips with a smaller tumor thickness. Therefore, no statement can be made about the applicability of the SLNB for tumors with a smaller tumor thickness.

The present study is a retrospective analysis, which also leads to limitations for cofounders and covariates. In particular, it should be noted that a relatively smaller number of patients with SLNB were analyzed than patients in the control group. This is due to the retrospective design and the real-world setting of this data set. Further, there was no re-evaluation or reprocessing of the histological blocks by a higher number of sections or additional staining, since the present work is intended to reflect a real-world setting. A re-examination might reduce the false negative rate, as could be shown analogously for melanoma.

We refrained from presenting a Kaplan–Meier curve for the SLNB result, as the number of positive SLNB results was too small in relation to the negative results to display a valid curve.

## Conclusion

It is possible to evaluate the lymph node status in patients’ cSCC by SLNB. Due to the frequent localization of these tumors at the head and face area with a complex lymphatic situation as well as possible changes of the lymph drainage caused by previous surgeries, the detectability of the sentinel lymph node is limited. The proportion of false-negative sentinel lymph nodes was high in the present study; only one patient with lymph node metastases in the course of the study already showed metastasis of the sentinel lymph node. While in melanoma it could be shown that wide local excision with subsequent linear repair does not have any impact on subsequent SLNB, there are only few data available for defect closure with local flaps [11]. However, the influence of surgery on lymphatic drainage is unknown in cSCC.

A large proportion of our patients refused to undergo SLNB despite the presence of a high-risk tumor. There was no difference concerning tumor-specific deaths in the two investigated groups. The available data do not indicate that SLNB provides an advantage in survival or nodal control. Therefore, SLNB should only be considered for high-risk tumors and needs be assessed individually.
